# Proportion of US Counties and Population Served by Certified Community Behavioral Health Clinics

**DOI:** 10.1001/jamahealthforum.2024.3001

**Published:** 2024-10-04

**Authors:** Amanda I. Mauri, Nuannuan Xiang, Danielle R. Adams, Jonathan Purtle

**Affiliations:** 1 Department of Public Health Policy and Management, New York University School of Global Public Health, New York; 2 Department of Health Policy and Management, Columbia University Mailman School of Public Health, New York, New York; 3 School of Social Work, College of Health Sciences, University of Missouri, Columbia

## Abstract

This case series evaluates the proportion of US counties and the US population residing within an area served by a certified community behavioral health clinic (CCBHC) from October 2016, when the first CCBHCs opened, to June 2024.

## Introduction

Certified community behavioral health clinics (CCBHCs), organizations that fulfill federal criteria about mental health and substance use disorder care regardless of ability to pay, are a cornerstone of bipartisan strategies to improve behavioral health care availability and quality. There are 2 nonmutually exclusive CCBHC types: expansion CCBHCs and Medicaid CCBHCs. Expansion CCBHCs receive Substance Abuse and Mental Health Services Administration (SAMHSA) expansion grant funding. Medicaid CCBHCs primarily receive per diem or monthly bundled Medicaid payments. Non-Medicaid CCBHCs may still receive Medicaid reimbursement through fee-for-service or other payment mechanisms.

The federal government invested substantial resources to expand the number of CCBHCs, including more than $1.7 billion in expansion grants, and an estimated $8.5 billion between 2022 and 2032 to expand CCBHC Medicaid bundled payments (eAppendix 1 in Supplement 1).^1^ Although these investments increased the number of CCBHCs, whether this expansion concentrates in specific areas or is dispersed throughout the country is unclear. This study analyzes CCBHC availability longitudinally, measuring the proportion of US counties and US population residing within a CCBHC service area between October 2016, when the first CCBHCs opened, and June 2024.

## Methods

We collected CCBHC county-level service area data for the 2 CCBHC types using government data, resulting in a comprehensive, exhaustive dataset of CCBHC service areas. The National Council for Mental Wellbeing (NCMW) reviewed and approved the final dataset. New York University’s institutional review board exempted this study from review and waived informed consent, as the data were deidentified. We followed the reporting guideline for case series.

Medicaid CCBHCs exist in the 12 states where the Centers for Medicare & Medicaid Services have approved, and states have implemented, CCBHC Medicaid bundled payments. We consulted state-specific resources to identify service areas (eAppendix 2 in Supplement 1). NCMW supplied information regarding when each clinic began and, in some cases, stopped receiving this payment.

For expansion CCBHCs, the primary data source was the SAMHSA Grants Dashboard, which provides award start and end dates and descriptions containing information on county-level CCBHC service areas. Sixty-one of all expansion grants (6.76%) were missing data on county-level service areas in the dashboard, so we consulted secondary materials (eAppendix 2 in Supplement 1).

The final dataset captured whether a county was served by an expansion CCBHC, Medicaid CCBHC, or any CCBHC (expansion or Medicaid) for each month between October 2016 and June 2024. Although the dataset did not capture actual population served, we used county-level service areas to measure the clinic's target geographic reach, as done in other studies.^1,2^ More than 50% of CCBHCs serve multiple counties, so measuring CCBHC availability using one service site would risk underestimating CCBHCs’ geographic impact.^3^ Analyses were conducted with STATA, version 18.0 (StataCorp).

## Results

**Figure 1.**
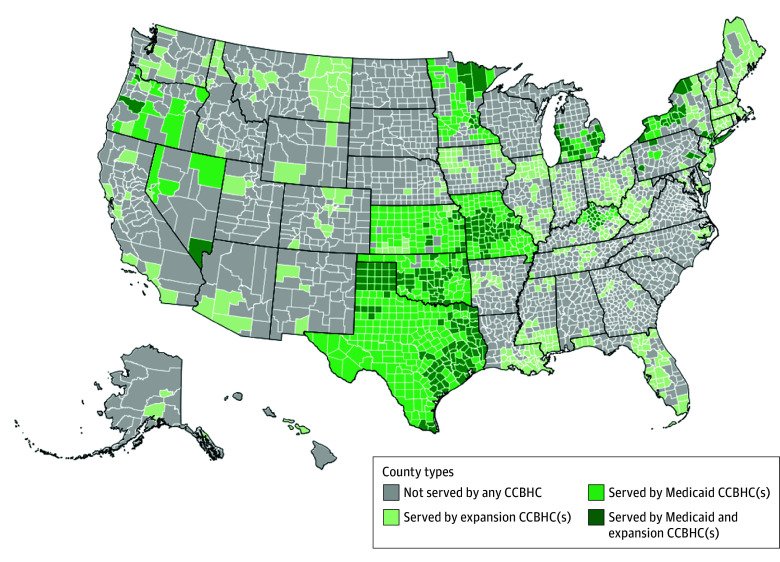
US Counties Served by Certified Community Behavioral Health Clinics (CCBHCs) As of June 30, 2024, the US states that approved and implemented CCBHC Medicaid bundled payments were Kansas, Kentucky, Michigan, Minnesota, Missouri, Nevada, New Jersey, New York, Oklahoma, Oregon, Pennsylvania, and Texas. On June 4, 2024, the Centers for Medicare & Medicaid Services added 10 new states to the Section 223 Medicaid Demonstration, which authorizes a per diem or monthly Medicaid prospective payment system for selected CCBHCs in participating states. The newly added states are Alabama, Illinois, Indiana, Iowa, Kansas, Maine, New Hampshire, New Mexico, Rhode Island, and Vermont. The start date for the Medicaid prospective payment varies by state; the first state will begin in July 2024, and others will launch as late as July 2025.

**Figure 2.**
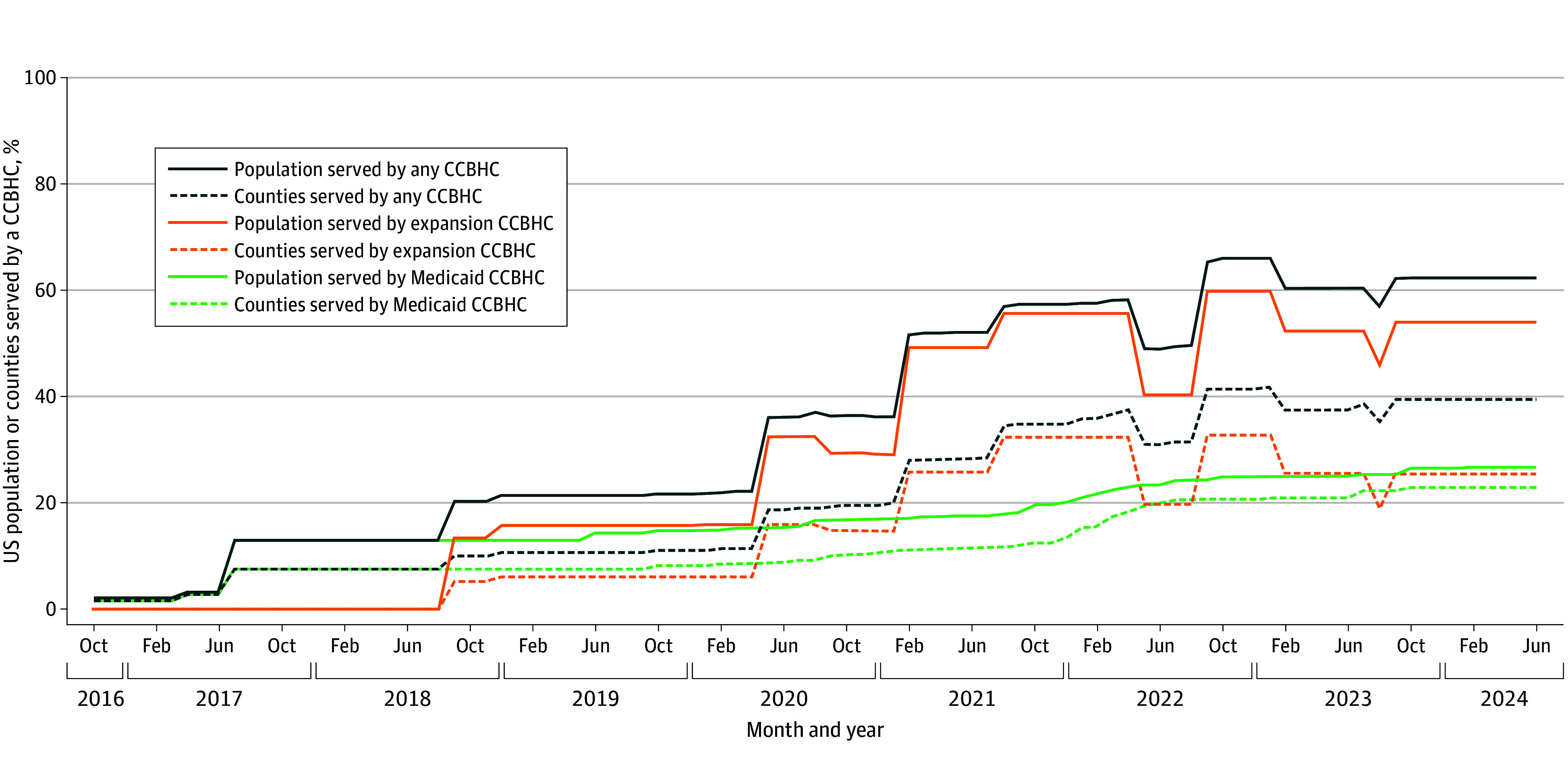
Proportion of US Counties and Population Residing Within a Certified Community Behavioral Health Clinic (CCBHC) Service Area The figure depicts the proportions on the last day of each month from October 31, 2016, to June 30, 2024. County population size was gathered from the US Census Bureau. The denominator used to calculate the proportion of counties and population for any CCBHC, expansion CCBHC, and Medicaid CCBHC equaled the total number of counties in the US and the total US population. Although Medicaid CCBHCs existed in only 12 states during the study period, the proportion of all counties and the US population residing within a Medicaid CCBHC service area was calculated using the total number of US counties and the total US population. These denominators were selected, rather than the sum of all counties or populations within states with a CCBHC Medicaid bundled payment to communicate the proportion of the total US population with access to a Medicaid CCBHC, regardless of whether the state implemented a CCBHC Medicaid bundled payment.

Figure 1 displays the county-level CCBHC geographic distribution by type as of June 30, 2024. The mean (SD) proportion of US counties and population residing within any service area across states was 39.43% (33.34%) and 55.66% (32.43%), respectively. When the first CCBHCs opened in October 2016, only 1.53% and 1.99% of counties and population, respectively, fell within a Medicaid CCBHC service area (Figure 2). By June 2024, these percentages grew to 22.85% and 26.63%, respectively. Although only 5.22% and 13.36% of counties and population, respectively, were within an expansion CCBHC service area in September 2018 (when the first expansion awards began), these proportions increased to 25.37% and 53.93% by June 2024.

## Discussion

This study suggests the proportion of US counties and population residing within a CCBHC service area increased over time; however, availability differences remained by geography and CCBHC type. One limitation was that our dataset did not capture the actual population the CCBHC served; as other studies have done, we used county-level service areas as a proxy for the clinic’s target geographic reach.^1,2^ CCBHCs offer more services than other community mental health centers and often add services and staff to fulfill CCBHC criteria.^2,3,4,5,6^ Therefore, expanding CCBHCs’ geographic reach may benefit public health by improving behavioral health care availability.

## Notes

Supplement 1. eAppendix 1. Timeline of Significant CCBHC Policy Developments eAppendix 2. Data Collection Process for County-Level CCBHC Service Areas

Supplement 2. Data Sharing Statement
