# Supplementary material for: Proportion of US Counties and Population Served by Certified Community Behavioral Health Clinics
Source: JAMA Health Forum. 2024 Oct 4;5(10):e243001. doi: 10.1001/jamahealthforum.2024.3001 (PMC11452811; doi:10.1001/jamahealthforum.2024.3001)
Supplement: Supplement 1. — eAppendix 1. Timeline of Significant CCBHC Policy Developments eAppendix 2. Data Collection Process for County-Level CCBHC Service Areas [file jamahealthforum-e243001-s001.pdf]

Supplemental Online Content

Mauri AI, Xiang N, Adams DR, Purtle J. Proportion of US counties and population served by certified community behavioral health clinics. *JAMA Health Forum*. 2024;5(10):e243001. doi:10.1001/jamahealthforum.2024.3001

eAppendix 1. Timeline of Significant CCBHC Policy Developments
eAppendix 2. Data Collection Process for County-Level CCBHC Service Areas

This supplemental material has been provided by the authors to give readers additional information about their work.

© 2024 Mauri AI et al. *JAMA Health Forum.*

eAppendix 1. Timeline of Significant CCBHC Policy Developments

Date	Event
April 2014	The Excellence in Mental Health Act, which encoded the CCBHC requirements in federal statute and created the Section 223 Medicaid Demonstration, became law as a part of the Protecting Access to Medicare Act (Pub. Law 113-93).
October 2016	The first Texas Certified Community Behavioral Health Clinics (T-CCBHC) opened.
April 2017	CCBHCs began participating in the Section 223 Medicaid Demonstration in Oklahoma and Oregon.
July 2017	CCBHCs began participating in the Section 223 Medicaid Demonstration in Minnesota, Missouri, Nevada, New Jersey, New York, and Pennsylvania.
September 2018	The Substance Abuse and Mental Health Services Administration (SAMHSA) awarded 64 Expansion grants under Notice of Funding Opportunity (NOFO) SM-18-019.
June 2019	The Centers for Medicare and Medicaid Services (CMS) approved Missouri and Oklahoma's state plan amendments for Medicaid to cover CCBHCs and reimburse CCBHCs using a bundled payment.<br><br>Pennsylvania withdrew from the Section 223 Demonstration, eventually converting all CCBHCs to a PA specific-model –Integrated Care and Wellness Clinics – through a Medicaid waiver.
March 2020	The CARES Act (Pub. Law 116-136) extended the Section 223 Medicaid Demonstration by 2 states.
May 2020	SAMHSA awarded 300 CCBHC Expansion grants under NOFO SM-20-012.
June 2021	CMS approved Oklahoma's state plan amendment for Medicaid to cover CCBHCs and reimburse CCBHCs using a bundled payment.
August 2021	SAMHSA awarded 102 CCBHC Expansion grants under NOFO SM-21-013.
October 2021	CCBHCs began participating in the Section 223 Medicaid Demonstration in Michigan.
January 2022	CCBHCs began participating in the Section 223 Medicaid Demonstration in Kentucky.
June 2022	The Bipartisan Safer Communities Act (Pub. Law 117-159) authorized the extension of the Section 223 Medicaid Demonstration by 10 states every 2 years.

© 2024 Mauri AI et al. *JAMA Health Forum.*

July 2022	CMS approved Kansas's state plan amendment for Medicaid to cover CCBHCs and reimburse CCBHCs using a bundled payment.
September 2022	SAMSHA awarded 301 CCBHC Expansion Grants under NOFOs SM-22-002 and SM-22-012.
September 2023	SAMHSA awarded 135 CCBHC Expansion Grants under NOFOs SM-23-016 and SM-23-024.
June 4, 2024	CMS added 10 new states – Alabama, Illinois, Indiana, Iowa, Kansas, Maine, New Hampshire, New Mexico, Rhode Island and Vermont – to the Section 223 Medicaid Demonstration.

eAppendix 2. Data Collection Process for County-Level CCBHC Service Areas

This appendix provides a detailed description of how we collected county-level CCBHC service areas. Organizations designated as a CCBHC may participate in two categories of CCBHC initiatives: (1) the CCBHC Expansion Grant Program and (2) CCBHC Medicaid programs. Because these initiatives are independent, we used different strategies to identify county-level service areas for Expansion CCBHCs and Medicaid CCBHCs. The National Council for Mental Wellbeing, a national organization representing CCBHCs, reviewed and approved the final dataset.

Step 1: CCBHC Expansion Grant Program

First, we gathered the county(ies) served by organizations that participated in the CCBHC Expansion Grant program. SAMHSA publishes descriptions of all awards associated with the seven Expansion Grant Notice of Funding Opportunities (NOFO) - SM-18-019, SM-20-012, SM-21-013, SM-22-002, SM-22-012, SM-23-016, and SM-23-024 - in the SAMHSA Grants Dashboard. We used a python script to download a list of these awards and their award descriptions as a CSV file. The script also used a search function to identify all county names from the descriptions and automatically added these to the dataset. The small proportion of descriptions that did not contain a county name were left blank. One researcher manually checked the county(ies) attributed to each award for correctness.

Of the 902 Expansion Awards, 84.59% (763) listed all county(ies) served by the organization receiving the grant. The award descriptions of another 78 awards (8.65%) did not explicitly list but implied the county(ies) served. For example, an award description stated that an organization "…will provide CCBHC services to children and adults in 6 Ohio counties …" We identified the 6 counties mentioned in the award description using another source, such as the organization or the relevant state Department of Mental Health's websites. Only 61 awards (6.76%) contained no geographic information. To identify the county(ies) served by these awards, we relied on four sources: a 2022 National Council for Mental Wellbeing survey of CCBHCs which asked respondents which county(ies) they served in 2022, other Expansion grants associated with the same organization, state department of health and mental health websites, and other geographic information provided in the SAMHSA Grants Dashboard.

Step 2: CCBHC Medicaid Initiatives

Second, we gathered the county(ies) served by organizations that receive(d) a CCBHC Medicaid bundled payment. State entities select which organizations participate in CCBHC Medicaid programs. Consequently, there is no national data source that contains information on the service areas for Medicaid CCBHCs. Thus, to gather county-level CCBHC service areas for the approximately 200 clinics that receive(d) a CCBHC Medicaid bundled payment in the 12 states with an authorized and implemented payment between October 16, 2016 and June 30, 2024, we relied on state-specific resources available in eAppendix 2 Exhibit 1. Some of the sources are publicly available on a state website (e.g., Kansas, Minnesota, and Texas). In other states (e.g., Michigan, Nevada,

© 2024 Mauri AI et al. *JAMA Health Forum.*

and Pennsylvania), there is no public resource providing information on CCBHC service areas. For these states, we partnered with the National Council for Mental Wellbeing. Specifically, a National Council for Mental Wellbeing staff member corresponded with relevant state representatives. The state representative then provided the National Council and our team with the county(ies) served for each entity in their state that received the authorized CCBHC Medicaid payment.

Exhibit 1. State-Specific Resources Used to Identify County(ies) Served by Medicaid CCBHCs

**State**	**Source**
Kansas	Link
Kentucky	Link 1, link 2, link 3, 2022 National Council for Mental Wellbeing Impact Survey
Michigan	Correspondence with Behavioral Health Program Specialist, CCBHC Demonstration, Michigan Department of Health and Human Services
Minnesota	Link – Under Providers Tab
Missouri	Link – Under General Information
New Jersey	Correspondence with Senior Health Policy Analyst at New Jersey Association of Mental Health & Addiction Agencies, Inc.
Nevada	Correspondence with by Social Services Program Specialist III, Nevada Department of Health and Human Services
New York	Correspondence with OMH CCBHC Project Team, NYS Office of Mental Health
Oregon	Link – Evaluation of Oregon's CCBHC Program
Oklahoma	Link – Under Providers Tab
Pennsylvania	Correspondence with Quality Assurance Risk Management Coordinator, PA Department of Human Services, Office of Mental Health and Substance Abuse Services
Texas	Link, Correspondence with Director of Integrated Care Strategy, Texas Department of Health and Human Services

Abbreviation: CCBHC, certified community behavioral health clinic.

© 2024 Mauri AI et al. *JAMA Health Forum.*
