# Supplementary material for: Proportion of US Counties and Population Served by Certified Community Behavioral Health Clinics
Source: JAMA Health Forum. 2024 Oct 4;5(10):e243001. doi: 10.1001/jamahealthforum.2024.3001 (PMC11452811; doi:10.1001/jamahealthforum.2024.3001)
Supplement: Supplement 2. — Data Sharing Statement [file jamahealthforum-e243001-s002.pdf]

Data Sharing Statement

Mauri. Proportion of US Counties and Population Served by Certified Community Behavioral Health Clinics. *JAMA Health Forum*. Published October 04, 2024.
doi:10.1001/jamahealthforum.2024.3001

Data
Data available: No
